# Books and Pamphlets Received

**Published:** 1893-02

**Authors:** 


### BOOKS AND PAMPHLETS RECEIVED.

"An American Text-Book of Surgery," by W. M. Keene, M. D., LL. D., and J. Wm. White, M. D., Ph. D. Published by W. B. Saunders. Philadelphia.

"Text-Book of Ophthalmology," by Dr. Ernest Fuchs. D. Appleton & Co., Publishers. New York.

"Naphy's Modern Therapeutics," volume I. By Allen J. Smith, M. D., and J. Aubrey Davis, M. D. Published by P. Blakiston, Son & Co. Philadelphia.

"Tuberculosis of the Bones and Joints," by N. Senn. M. D. F. A. Davis Co., Publishers. Philadelphia.

"Swanzy on the Eye." Published by P. Blakiston, Son & Co. Philadelphia.

"Addresses and Essays," by G. Frank Lydston, M. D. Published by Renz & Henry. Louisville, Ky.

"Medical Jurisprudence and Toxicology." By H. C. Chapman, M. D. W. B. Saunders, Philadelphia, Publisher.

"Notes on the Newer Remedies," by David Cerna, M. D., Ph. D. Published by W. B. Saunders. Philadelphia.

"Kirke's Handbook of Physiology." P. Blakiston, Son & Co., Publishers.

"Essentials of Nervous Diseases and Insanity." By John C. Shaw, M. D. Published by W. B. Saunders. Philadelphia.

"Gower's Syphilis and Nervous System." P. Blakiston, Son & Co., Philadelphia, Publishers.

"Kleen's Handbook of Massage." Published by P. Blakiston, Son & Co. Philadelphia.

"Diseases of Lungs, Heart and Kindneys." By N. S. Davis, M. D. Published by F. A. Davis & Co. Philadelphia.

"The Principles and Practice of Bandaging," By G. G. Davis, M. D. Published by Geo. S. Davis. Detroit.

